# Exercise and Beta-Glucan Consumption (*Saccharomyces cerevisiae*) Improve the Metabolic Profile and Reduce the Atherogenic Index in Type 2 Diabetic Rats (HFD/STZ)

**DOI:** 10.3390/nu8120792

**Published:** 2016-12-17

**Authors:** Eric Francelino Andrade, Andressa Ribeiro Veiga Lima, Ingrid Edwiges Nunes, Débora Ribeiro Orlando, Paula Novato Gondim, Márcio Gilberto Zangeronimo, Fernando Henrique Ferrari Alves, Luciano José Pereira

**Affiliations:** 1 Department of Veterinary Medicine, Federal University of Lavras, Mail Box 3037, Lavras 37200-000, Brazil; pngondim@hotmail.com (P.N.G.); zangeronimo@prpg.ufla.br (M.G.Z.); 2 Department of Health Sciences, Federal University of Lavras, Mail Box 3037, Lavras 37200-000, Brazil; andressaveigalima@outlook.com (A.R.V.L.); fernando.ferrari@dsa.ufla.br (F.H.F.A.); lucianojosepereira@dsa.ufla.br (L.J.P.); 3 Department of Animal Sciences, Federal University of Lavras, Mail Box 3037, Lavras 37200-000, Brazil; inunes2611@gmail.com; 4 Department of Agricultural Sciences, Federal University of Jequitinhonha and Mucuri Valleys, Rua Vereador João Narciso, 1380—Bairro Cachoeira, Unaí 3861-000, Brazil; debora.ribeiro@ufvjm.edu.br

**Keywords:** dietary fibers, glycemic control, metabolic profile

## Abstract

Physical activity and the ingestion of dietary fiber are non-drug alternatives commonly used as adjuvants to glycemic control in diabetic individuals. Among these fibers, we can highlight beta-glucans. However, few studies have compared isolated and synergic effects of physical exercise and beta-glucan ingestion, especially in type 2 diabetic rats. Therefore, we evaluated the effects beta-glucan (*Saccharomyces cerevisiae*) consumption, associated or not to exercise, on metabolic parameters of diabetic Wistar rats. The diabetes *mellitus* (DM) was induced by high-fat diet (HFD) associated with a low dose of streptozotocin (STZ—35 mg/kg). Trained groups were submitted to eight weeks of exercise in aquatic environment. In the last 28 days of experiment, animals received 30 mg/kg/day of beta-glucan by gavage. Isolated use of beta-glucan decreased glucose levels in fasting, Glycated hemoglobin (HbA1c), triglycerides (TAG), total cholesterol (TC), low-density lipoprotein (LDL-C), the atherogenic index of plasma. Exercise alone also decreased blood glucose levels, HbA1c, and renal lesions. An additive effect for reducing the atherogenic index of plasma and renal lesions was observed when both treatments were combined. It was concluded that both beta-glucan and exercise improved metabolic parameters in type 2 (HFD/STZ) diabetic rats.

## 1. Introduction

Diabetes *mellitus* (DM) is a metabolic disorder characterized by chronic hyperglycemia, caused by the absence or reduction in insulin production (type 1 DM) as well as the resistance to the action of this hormone, featuring type 2 DM [1]. About 90% of DM cases are of type 2, and this fact is associated with increased incidence of obesity and obesity in the general population, especially in developing nations [2,3]. In addition, DM may predispose to diseases, such as retinopathy, nephropathy, neuropathy and heart disease, further aggravating the health condition of patients [2,4].

Glycemic control in diabetic patients can be achieved through the use of exogenous insulin and/oral hypoglycemic drugs [5]. However, the interaction between medications could cause side effects, and does not prevent the diseases associated with DM, making necessary the search for non-pharmacological alternatives to assist in the maintenance of blood sugar levels [4,6]. In this sense, the practice of physical exercise and diet therapy has been recommended as a treatment or therapeutic adjuvant [7]. Physical exercise increases the uptake and utilization of circulating glucose and improves insulin sensitivity [8]. The ingestion of some dietary fibers has also been reported to show antihyperglycemic action—mainly by reducing the absorption of carbohydrates and lipids in the intestine. Among these fibers, we can highlight beta-glucans that are polysaccharides found in the composition of cereal, fungi, bacteria and some grass cell walls [9].

The chemical structure of beta-glucan varies according to its origin [10]. Beta-glucans found in plants and cereals are linear and have branchings with β-1,3/1,4-type glycosidic linkages (soluble with low molecular weight), while those found in yeasts and fungi have β-1,3/1,6-type glycosidic linkages (insoluble with high molecular weight) [11]. These conformations make beta-glucans exhibit distinct physicochemical characteristics, such as molecular mass and solubility [12,13]. Cereal beta-glucans are reported to show metabolic potential, while those from fungi and yeast increase immune response [10,14,15]. Although fungi beta-glucans are recognized to modulate the immune response [16], recent studies from our group have also demonstrated interesting metabolic effects of yeast beta-glucans (*Saccharomyces cereviseae*) [10,17,18].

Considering the previously known effects of both exercise and the beta-glucan on glycemic control and metabolism, it is necessary to investigate the concomitant action of these agents in the treatment of type DM. In addition, there is a shortage of studies evaluating such effects in type 2 diabetes model. Thus, the present study aimed to evaluate the effects of beta-glucan (*Saccharomyces cerevisiae*), associated or not to physical exercise, on the metabolic parameters of type 2 diabetic rats (HFD/STZ).

## 2. Materials and Methods

### 2.1. Animals

This study was approved by the Ethics Committee on Animal Use of Federal University of Lavras (CEUA protocol 002/2015). The animals were kept in accordance with the Guide to the Care and Use of Experimental Animals (1993). The number of animals per group was kept at a minimum for ethical reasons but still enough to reach statistical significance. Thus, a power calculation test was performed to determine the sample size. The sample size was determined to provide 80% power to recognize a significant difference of 20% among groups and a standard deviation of 15% with a 95% confidence interval (α = 0.05).

We used adult male Wistar rats (*Rattus norvegicus albinos*)—from the Animal Laboratory of the Federal University of Lavras (UFLA). Animals weighed 195.0 ± 15.7 g at the beginning of the study. Initially, rats were submitted to seven days of acclimatization in polypropylene boxes (dimensions 41 cm × 34 cm × 17.5 cm), containing wood shavings (for absorbing urine and water). Six animals were placed in each box. Throughout the experimental period, the rodents remained under controlled temperature (22 ± 2 °C), humidity (45% ± 15%) and luminosity (12–12 h light-dark cycle) conditions. High-fat diet and water were provided ad libitum throughout the experiment. 

### 2.2. Induction of Diabetes Mellitus

At the end of the acclimatization period, all animals were submitted to type 2 diabetes induction protocol as described by Wang et al. [19]. The animals received high-fat diet (HFD—25% fat, 48% carbohydrates and 20% protein) for 28 days. Then, a low dose of streptozotocin (dissolved in citrate buffer—pH = 4.5) was injected intraperitoneally (STZ—35 mg/kg). Blood glucose levels were measured 48 h after STZ injection. Rats with blood glucose levels above 200 mg/dL [19] were considered diabetic. This model mimic advanced stages of type 2 diabetes in humans [19,20]. Rats that did not reach these glucose values were excluded from the experiment. Glycemia was checked weekly to ensure that diabetes was not reversed.

After diabetes induction, animals were randomly divided into four groups containing six animals each. A completely randomized experimental design in a 2 × 2 factorial scheme was used: with or without exercise and with or without beta-glucan.

### 2.3. Physical Training

After an acclimatization period, an adaptation to the aquatic environment was performed. Animals undergoing physical training remained for two hours daily, during seven days, in a polyethylene tank with a total capacity of 300 L, containing five centimeters of water at a temperature of approximately 32 ± 2 °C. The purpose of this acclimatization was to reduce stress against the aquatic environment, without causing, however, changes arising from the physical training [21].

In the following week, animals were submitted to progressive swimming sessions with time increments. This phase consisted of swimming without load, in 50 cm of water (in order to avoid animal tail contact with the bottom of the tank), where the animals swam 10 min in the first day, increasing 10 min daily until the end of six days, when each animal was swimming for 60 uninterrupted minutes without load [22].

In the subsequent eight weeks, the animals swam for 60 min daily, five times a week with a load of 5% of their body weight. This load causes improvement in the animals’ endurance capacity, characterizing moderate intensity aerobic exercise [22]. After training sessions, we dried the animals with absorbent towels, before returning them to their cages [21].

### 2.4. Administration of Beta-Glucans

Simultaneously with training, in the last 28 days of the experiment, the animals in beta-glucan groups received a experimental solution and controls received saline—both by gavage. Beta-glucan solutions that contained 30 mg/kg of powder diluted in 0.3 mL saline solution prepared daily.

Beta-glucan used in the present study were derived from yeast *Saccharomyces cerevisiae*, with structural β-1,3/1,6 conformation. The beta-glucan powder presented the following composition: β-glucans—Min. 60.0%; Crude Protein—Max. 8.0%; pH (solution 2%) 4.0–7.0; Ash—Max. 10.0 g/100 g. Distribution of particle size: mean—41 μm; <20 μm 19%; 20–50 μm 43%; 50–100 μm 28%; 100–200 μm 10%; >200 μm 0%; Fluidity (seconds)—70.2; Angle of repose (degrees) 31.2; Compressibility 37%; Water retention capacity (mean) 7.4; and Solubility rate in water 7.9. The solutions were always administered daily in the morning. In animals under physical training, gavage was always performed with a minimum of 45 min before exercise, as described in previous studies [21,23].

### 2.5. Collection of Biological Material and Assessment of the Atherogenic Index of Plasma

At the end of the experimental period (eight weeks), the animals fasted for eight hours. Euthanasia was conducted by cardiac puncture under anesthesia (sodium thiopental 50 mg/kg ip). Glycated hemoglobin (HbA1c) and other blood biochemical parameters such as glucose, triacylglycerols (TAG), high density lipoprotein (HDL-C) and total cholesterol (TC) were determined using commercial kits (Labtest Diagnostica^®^, Belo Horizonte, Brazil and Gold analyzes diagnoses^®^, Belo Horizonte, Brazil) as described by Amr and Abeer [24]. The low-density lipoprotein (LDL) + very-low-density lipoprotein cholesterol (VLDL-C) levels of each animal were obtained by using the following equation: total cholesterol − HDL-C = LDL + VLDL-C [25]. Additionally, the animals’ atherogenic index of plasma was calculated using the equation: log (TG)/(HDL-C), which is used as a significant predictor of atherosclerosis [26]. This index was used because type 2 diabetes increases one’s chances of developing atherosclerosis [27].

### 2.6. Lee Index Assessment and Chemical Composition of the Body

The Lee index was calculated dividing the cubic root of body weight (grams) by the naso-anal length (cm) [18,28]. Internal organs, skin, head, feet and tail were removed from the animals and the clean carcasses were weighed and processed. Percentages of water, protein, fat and mineral matter present in the carcasses were evaluated by the meat FoodScan™ NIR analyzer (near-infra-red) (Foss, Warrington, UK) as performed by Vickers et al. [29]. This evaluation method of carcass composition has been considered as the gold standard [29].

### 2.7. Histological Analysis

Fragments of the right kidney and liver were fixed in 10% buffered formalin for 48 h, and then processed routinely for preparation of histological slices, which were then colored with hematoxylin-eosin [30]. An experienced veterinary pathologist conducted all histopathologic analysis (blind about experimental treatments). Tissue integrity, as well as the presence of alterations, were considered in the evaluations. Liver tissue ratings were assigned according to the presence and/or degree of steatosis as follows: no change—1; discreet—2; light—3; moderate—4; and severe—5. Steatosis was classified according to the presence of vacuoles in hepatocytes. Staining was performed with Periodic acid-Schiff (PAS) indicating accumulation of lipids or glycogen.

Similarly, the presence of renal lesions was scored as: no change—1; mild degeneration—2; low degeneration—3; moderate degeneration—4; marked degeneration 5. We observed the presence of alterations in the proximal and distal convoluted tubules, and the presence of calcifications in the glomerulus.

### 2.8. Statistical Analysis

Data were subjected to analysis of variance (two-way ANOVA) and means were compared by Tukey test (*p* < 0.05). Nonparametric data of liver and kidney damage scores were analyzed by the Kruskal–Wallis test (*p* < 0.05). We performed all analyses using statistical program Sisvar (version 5.3, Universidade Federal de Lavras, Lavras, Minas Gerais, Brazil) [31].

## 3. Results

Animals submitted to physical training, or consuming beta-glucan isolated and in association, presented lower fast blood glucose and HbA1c levels than diabetic animals (Table 1). Serum levels of TAG, TC and LDL-C were significantly reduced in animals consuming beta-glucan, independently of physical training. In addition, HDL-C levels were higher in animals treated with beta-glucan. Exercise did not significantly alter this parameter (Table 1). The atherogenic index of plasma in animals treated with beta-glucan was lower in comparison to without treatment. An additive effect of beta-glucan and physical exercise was observed for the atherogenic index of plasma. Blood parameters and atherogenic index of plasma means and standard deviations are presented in Table 1.

**Figure 1:**
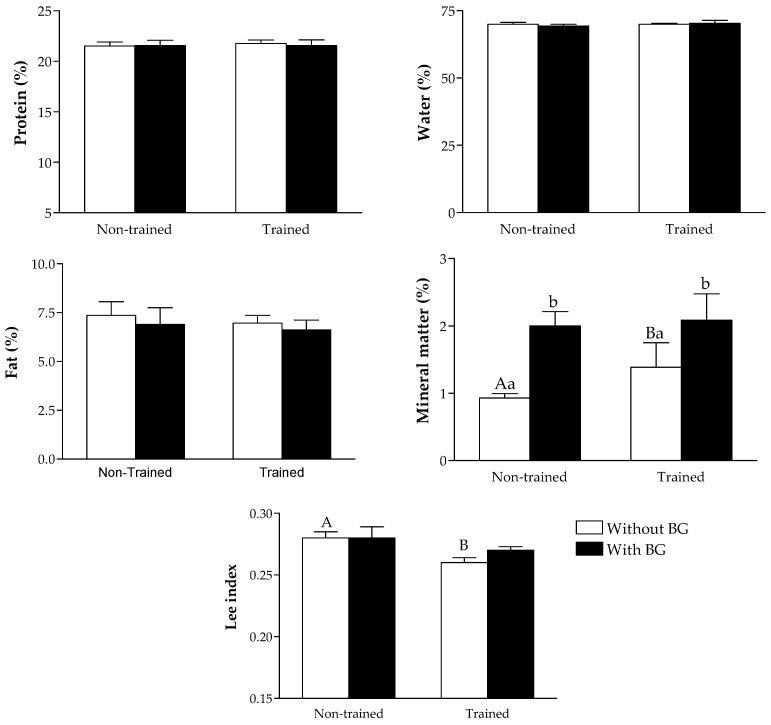
Chemical body composition (water, protein, fat and mineral matter) and Lee index of type 2 diabetic rats (high-fat diet/streptozotocin) submitted to physical training and treated with beta-glucan (30 mg/kg/day). ^A,B^ Significant difference between trained and non-trained groups; ^a,b^ Significant difference between groups with and without beta-glucans.

All treatments promoted similar results in the percentage of protein, fat and water in animals’ carcasses. An increase in the percentage of mineral matter was observed in groups under physical training and beta-glucan consumption, with an extra increase when both treatments were associated. Exercise promoted a decrease in the Lee index compared to controls, with similar results among the other groups (Figure 1).

**Figure 2:**
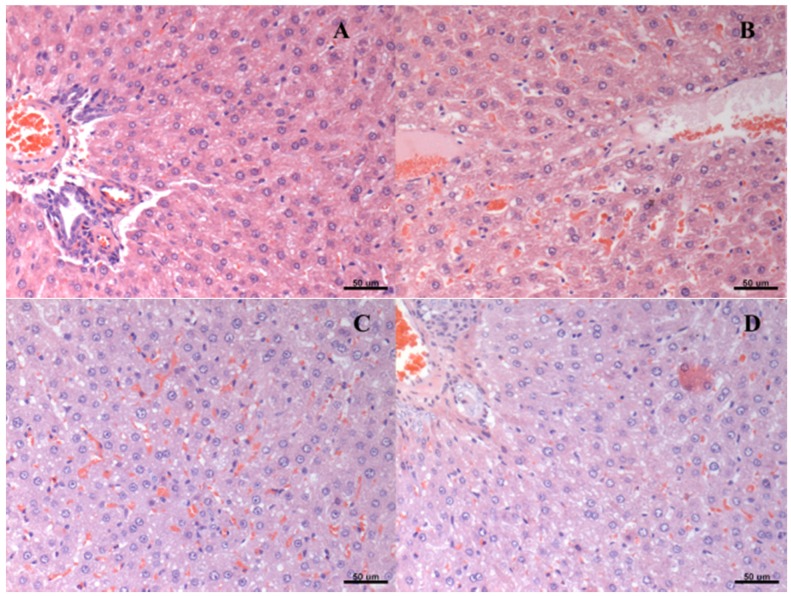
Histological representation (hematoxylin and eosin—20×) of degrees of hepatic steatosis in type 2 diabetic rats (HFD/STZ) submitted to physical training and/or treated with beta-glucans (30 mg/kg/day). (**A**) diabetes *mellitus*; (**B**) diabetes *mellitus* + beta-glucan; (**C**) diabetes *mellitus* + exercise; (**D**) diabetes *mellitus* + beta-glucan + exercise.

**Figure 3:**
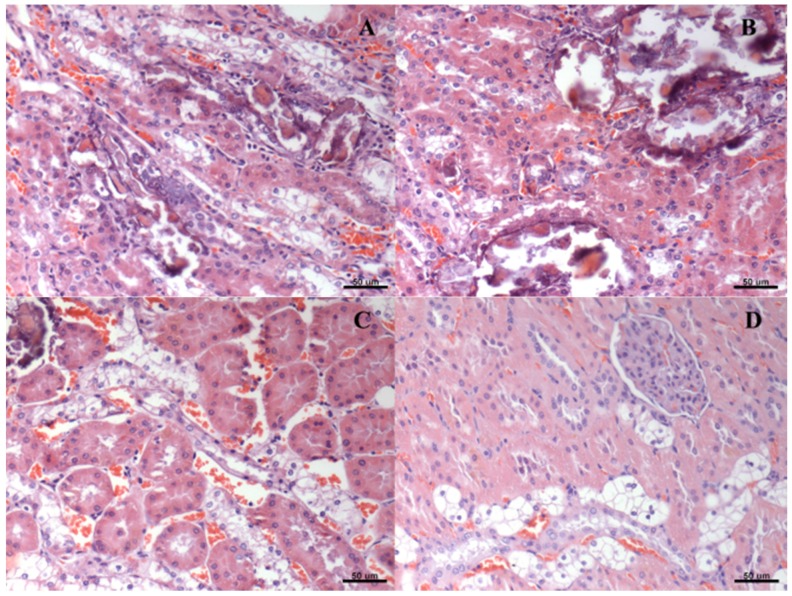
Histological representation (hematoxylin and eosin—20×) of degrees of renal degeneration in type 2 diabetic rats (HFD/STZ) submitted to physical training and/or treated with beta-glucans (30 mg/kg/day). (**A**) diabetes *mellitus*; (**B**) diabetes *mellitus* + beta-glucan; (**C**) diabetes *mellitus* + exercise; (**D**) diabetes *mellitus* + beta-glucan + exercise.

Liver histopathology slices revealed similar signs of steatosis in all groups (Table 2). Likewise, hydropic degeneration was found in the renal tissue from all groups. The degree of these lesions was attenuated by both physical exercise and beta-glucan ingestion (Table 3). Figure 2 and Figure 3 represent, respectively, hepatic steatosis and renal degeneration in the different experimental groups. 

## 4. Discussion

The main findings of this study were related to improved glycemic control and reduced predisposition to atherosclerosis in animals subjected to both exercise beta-glucan consumption. Moreover, circulating lipoproteins levels, such as total cholesterol, LDL-C, and HDL-C, were improved in animals consuming beta-glucan, independently of physical exercise.

The effects of physical exercise on the improvement of glycemic control in diabetic patients (decrease in HbA1c and fasting glucose) are frequently reported [32,33,34]. Generally, this effect is due to the increased glucose uptake by skeletal muscle during exercise and increased insulin sensitivity for some hours after physical activity [35]. A beneficial effect in the glycemic control, in our study, was also observed after beta-glucan ingestion, as reported elsewhere in both animal [10] and in human studies [36]. Blood glucose control by beta-glucan consumption is probably due to the fact that these fibers form a gelatinous barrier in the intestinal lumen, hindering the absorption of carbohydrates and lipids by enterocytes [10,37,38]. In this sense, the same mechanism can be used to justify a reduction in circulating levels of total cholesterol, LDL-C and TAG found in groups treated with beta-glucan, with and without exercise. The improvement of the lipid profile, despite the consumption of beta-glucan, was a feature also observed in previous studies from our group [10,17]. The lower lipid absorption in the intestine favors the use of excessive cholesterol to the formation of bile salts in the liver, causing decreased blood concentrations of total cholesterol and LDL-C [39]. This mechanism is generally used to explain the anti-hypercholesterolemic effect of dietary fibers [39].

Among the possible beta-glucan’s action, we can highlight the stimulation of intestinal motility, as well as changes in the microbiota and modulation of hormones secretion in the intestine [27,40,41]. Intestinal motility can be stimulated by the increase in the viscosity of the digesta in the lumen, due to the formation of a gelatinous layer [42,43]. In addition, beta-glucan decreases carbohydrates and lipid absorption [42], and consequently decreasing constipation problems [11]. High molecular weight beta-glucans (1,3/1,6) can also serve as a substrate for symbiotic microorganisms present in the intestine, increasing IgA and lysozyme secretion, and, as a consequence, immune resistance [40]. Another mechanism related to the functional effect of beta-glucans is satiety, mediated by gastrointestinal hormones [41]. Beta-glucans modulate the secretion of ghrelin and peptide YY, in order to inhibit hunger, acting indirectly in glycemic and lipidemic control [44].

In this study, we did not observe reduction of circulating lipids in trained animals. This may be related to the training time or duration of exercise sessions. Moura et al. [45] also found similar levels of HDL-C, LDL-C and TAG in diabetic rats (induced by alloxan) and subjected to 44 days of training, compared to sedentary diabetic rats. Another study demonstrated that twelve weeks of aquatic training decreased cholesterol levels and TAG in diabetic Zucker rats [46]. However, in the present study, no significant differences were observed in circulating lipids in animals submitted to physical training, and the atherosclerotic plasma index was reduced when there was an association of exercise and beta-glucan consumption. This additive effect may be related to improvement of the lipid profile provided by the dietary fiber [47], and to the recognized cardiovascular benefits of exercise [48].

Liver and renal lesions observed in all groups are consistent with those observed in type 2 diabetes, where circulating lipid levels promote increased fat deposition both in the liver and kidney [49,50]. However, even with the benefits observed with beta-glucan consumption or exercise, no changes were found in the degree of steatosis in any treatment. Beta-glucan consumption did not significantly alter steatosis either in a recent study of our group that investigated the effects of these fibers in rats submitted to high-fat diet [18]. On the other hand, it was observed that, in Sprague–Dawley rats, hepatic steatosis was reversed after eight weeks of treadmill exercise associated with restrictive diet (low-fat) [51]. Thus, it is possible that, in this study, steatosis was not attenuated because the animals were consuming a high-fat diet throughout the experimental period.

Regarding the effects of exercise, with or without the beta-glucan on the attenuation of renal lesions, it can be considered two mechanisms. The first one involves the reduction in lipotoxicity against moderate exercise [52], since the oxidative stress observed in diabetic patients is one of the factors that predispose to kidney damage [53]. The second one, more likely to explain the results of the present study, is the fact that exercise in moderate intensity promotes improvement in glycemic control, which consequently reduces the generation of advanced glycation-end products (AGE) [54]. Thus, the higher the blood glucose levels, the higher the formation of AGE that attack the kidney tissue and cause diabetic nephropathy [55].

Results of the present research show very promising effects of beta-glucan ingestion for glucose control. Complimentary studies are encouraged, evaluating insulin/leptin levels and inflammatory and cardiovascular parameters as well. The improvement of metabolic parameters in animals that consumed beta-glucans may be related to a decrease in the absorption of nutrients that increase plasma levels of glucose and lipids [37,39]. These changes were not as evident in animals subjected to exercise, possibly due to high-fat diet maintenance for the entire period.

## 5. Conclusions

Both exercise and beta-glucan consumption alone improved glycemic control in diabetic rats. In the present study, the combination of exercise and beta-glucans improved the atherosclerotic index and decreased renal lesions when compared to the isolated use of the treatments.

## Figures and Tables

**Table 1:** Biochemical parameters and atherogenic index of plasma in type 2 diabetic rats (high-fat diet/streptozotocin) submitted to physical training and treated beta-glucan (30 mg/kg/day).

	Beta-Glucan	Physical Training	
		Without	With
Glucose (mg/dL)	Without	371.0 (±21.4) ^A,a^	335.0 (±10.4) ^b^
	With	311.0 (±25.0) ^b^	327.5 (±42.6)
HbA1c (mg/dL)	Without	9.4 (±0.4) ^A,a^	8.8 (±0.3) ^B^
	With	8.33 (±0.1) ^b^	8.8 (±0.4)
Triacylglycerols (mg/dL)	Without	105.8 (±11.1) ^a^	99.7 (±2.2) ^a^
	With	71.5 (±7.2) ^b^	57.5 (±12.8) ^b^
Total cholesterol (mg/dL)	Without	88.8 (±22.9) ^a^	85.4 (±9.4) ^a^
	With	65.1 (±3.3) ^b^	63.8 (±7.2) ^b^
HDL-C (mg/dL)	Without	34.33 (±3.8) ^a^	37.7 (±6.9)
	With	42.66 (±5.2) ^b^	44.26 (±4.0)
LDL-C (mg/dL)	Without	34.6 (±20.1) ^a^	33.4 (±5.8) ^a^
	With	10.95 (±3.4) ^b^	19.6 (±4.9) ^b^
Atherogenic index of plasma	Without	1.6 (±0.6) ^a^	1.3 (±0.2) ^a^
	With	0.6 (±0.1) ^A,b^	0.4 (±0.1) ^B,b^

^a,b^ Means followed by different letters in columns indicate significant differences between groups with and without beta-glucan treatment (*p* < 0.05); ^A,B^ Means followed by different letters in lines indicate significant difference between groups with and without physical training (*p* < 0.05).

**Table 2:** Degree of hepatic steatosis in type 2 diabetic rats (HFD/STZ) submitted to physical training and/or treated with beta-glucans (30 mg/kg/day).

Group	Score of Steatosis				
	*	**	***	****	*****
A	0	3	0	3	0
B	1	3	2	0	0
C	2	3	0	1	0
D	1	5	0	0	0

* No change. ** Discreet Degeneration; *** Mild degeneration; **** Moderate degeneration; ***** Marked degeneration; A: diabetes *mellitus*; B: diabetes *mellitus* + beta-glucan; C: diabetes *mellitus* + exercise; D: diabetes *mellitus* + beta-glucan + exercise.

**Table 3:** Degree of renal degeneration in type 2 diabetic rats (HFD/STZ) submitted to physical training and/or treated with beta-glucans (30 mg/kg/day).

	Score of Renal Degeneration				
Group	*	**	***	****	*****
A	0	0	0	2	4
B	0	0	0	6	0
C	0	0	1	5	0
D ^#^	0	0	1	5	0

* No change; ** Discreet Degeneration; *** Mild degeneration; **** Moderate degeneration; ***** Marked degeneration; # Difference compared to the DM group; A: diabetes *mellitus*; B: diabetes *mellitus* + beta-glucan; C: diabetes *mellitus* + exercise; D: diabetes *mellitus* + beta-glucan + exercise.
